# Low Aspartate Aminotransferase/Alanine Aminotransferase Ratio as an Indicator of Metabolic Syndrome Among HIV Patients on Dolutegravir Therapy in Southwestern Uganda

**DOI:** 10.7759/cureus.77166

**Published:** 2025-01-08

**Authors:** Daniel Nzaramba, Charles Nkubi Bagenda, Hope Mudondo, Jazira Tumusiime, Elastus Ssemwanga, Darlington Muhwezi, Sylvia Achieng Lumumba, Ritah Kiconco, Simon Peter Rugera

**Affiliations:** 1 Department of Medical Laboratory Science, Mbarara University of Science and Technology, Mbarara, UGA; 2 School of Medical Laboratory Technology, Mayanja Memorial Medical Training Institute, Mbarara, UGA; 3 Municipal Health Department, Ibanda Municipality, Ibanda, UGA; 4 Department of Medical Laboratory Sciences, Technical University of Mombasa, Mombasa, KEN; 5 Biochemistry, Soroti University, Soroti, UGA

**Keywords:** metabolic syndrome (mets), predictive power, proportion, ratio, serum transaminases

## Abstract

Purpose: This study aimed to investigate the association between the aspartate aminotransferase-to-alanine aminotransferase (AST/ALT) ratio and metabolic syndrome (MetS) among HIV-infected patients on dolutegravir-based antiretroviral therapy (ART).

Patients and methods: A cross-sectional study was conducted on 377 adults on dolutegravir-based ART for at least one year. Data were collected from July 1 to August 15, 2024. Participants were systematically sampled, data were collected using a pre-tested questionnaire, anthropometric measurements were taken, and blood samples were collected for biochemical analysis. A low AST/ALT ratio was defined as ≤ 1 and MetS as the presence of at least three of the following: central obesity, fasting hyperglycemia, elevated triglycerides, low HDL-C, and hypertension (International Diabetes Federation (IDF) Consensus worldwide definition, 2006). Logistic regression was used to assess the association between low AST/ALT ratio and MetS, and receiver operating curve (ROC) analysis was conducted to evaluate its predictive performance.

Results: The median age of the participants was 44 years (interquartile range (IQR): 30-59), with 56.2% being female. The prevalence of MetS was 35.3% (133/377, 95%CI: 30.6-40.3). A significant association was found between low AST/ALT ratio and MetS (aOR: 2.19, 95% CI: 1.28-3.73, p = 0.004). Female gender (adjusted odds ratio (aOR): 3.68, 95% CI: 2.07-6.55, p < 0.001) and smoking (aOR: 3.96, 95% CI: 1.77-8.86, p < 0.001) were also significantly associated with MetS. The ALT/AST ratio had a significant predictive power for MetS (AUC = 0.583, 95% CI: 0.523-0.643).

Conclusion: The prevalence of MetS is high. A low AST/ALT ratio is significantly associated with MetS, making it a potential biomarker among HIV patients on ART.

## Introduction

To end the acquired immunodeficiency syndrome (AIDS) pandemic by 2030, the Joint United Nations Programme on HIV/AIDS (UNAIDS) announced the 95-95-95 plan in 2014 [1]. The goal was to have 95% of all people living with the human immunodeficiency virus (PLWHIV) diagnosed, 95% of those diagnosed to be receiving antiretroviral therapy (ART), and 95% of those treated to have their viral suppression intact [1]. By 2020, 84% of PLWHIV in the world were aware of their status, 87% were receiving ART, and 90% of these had viral suppression [2]. In Uganda, 89% were aware of their status, 84% of PLWHIV were enrolled in ART, and 90% were virally suppressed [3].

In 2018, Uganda adopted dolutegravir (DTG)-based ART as the first-line standard of care for PLWHIV, in accordance with recommendations from the World Health Organization (WHO) [3,4]. Dolutegravir is an integrase inhibitor that has a greater genetic resistance barrier, superior tolerability, reduced risk of medication interactions, and increased efficacy [5]. This medication is commonly taken with either tenofovir/lamivudine or abacavir/lamivudine, two nucleoside/tide reverse transcriptase inhibitors [4,6]. 

Dolutegravir-based ART has been associated with the consequent development of hyperglycemia [7], dyslipidemia, hypertension, and weight gain, which are components of metabolic syndrome (MetS) [8,9]. Several risk factors for cardiometabolism, including elevated blood pressure (BP), obesity, dyslipidemia, insulin resistance, and hyperglycemia, are collectively known as MetS [8]. Studies have established an association between DTG and metabolic complications that meet the MetS criteria [9,10,11,12,13,14].

MetS can cause necrosis, fat accumulation, and atypical hepatocyte metabolism [15-17]. In nonalcoholic fatty liver disease (NAFLD) and liver damage, enzymes of the liver that are typically detected in plasma, including aspartate transaminase (AST) and alanine transaminase (ALT), will pathologically increase [18]. Numerous studies revealed a strong relationship of ALT and AST, with MetS [8]. Indicators of hepatic dysfunction include ALT and AST in various conditions, including NAFLD, which is strongly associated with MetS [19]. Hepatic expression of MetS includes NAFLD and is related to obesity, dyslipidemia, insulin resistance, increased visceral fat, and lipoatrophy. MetS is a prerequisite for NAFLD, and elevated AST and ALT levels may be linked to NAFLD severity [20].

Early detection of MetS may aid in the prioritization of therapies aimed at lowering the risk of these cardiometabolic disorders and enhancing the long-term health of HIV-positive individuals [21]. According to a population-based cohort research conducted on Korean people aged 40-70, the AST/ALT ratio gives a reliable indicator of the development of MetS and its constituent parts in the future [22]. In addition, it is documented that the NAFLD can be inferred from the AST/ALT ratio, a feature of MetS, namely liver-related insulin resistance [22,23]. While elevated transaminase activity indicates liver disease, the prognosis and severity of liver injury are associated with the AST/ALT ratio [18]. A greater AST/ALT ratio indicates alcoholic liver disease, while a lower ratio indicates non-alcoholic steatohepatitis [8]. A study reported a link between a reduced AST/ALT ratio and having MetS [24,25].

For early detection, the AST/ALT ratio is utilized as a feature of MetS, and the availability of readily quantifiable blood markers related to MetS is important [26]. However, scant data support the association between the AST/ALT ratio and MetS, the predictive use of the AST/ALT ratio for MetS, and the components in HIV-positive individuals on dolutegravir-based ART.

Despite the high reported prevalence of individual metabolic derangements in Uganda, the magnitude of MetS and how it relates to the AST/ALT ratio in HIV-positive individuals on DTG-based ART has not been investigated, and hence the need to investigate this burden as MetS is associated with a higher risk of cardiovascular illnesses and diabetes mellitus than do its constituent parts [21] Therefore, this study determined the prevalence of MetS and its association with the AST/ALT ratio among HIV-positive patients on DTG-based ART.

## Materials and methods

Study design, site, and population

An analytical and descriptive cross-sectional study was conducted. The study was conducted at the ART Clinic of Ruhoko Health Centre IV, which is situated in Kanyakyeko Parish, Kagongo Division, approximately one kilometer from Ibanda Municipality in Ibanda District, South Western Uganda. The facility is 74 kilometers from Mbarara City, accessible via Ibanda Road. Ruhoko Health Centre IV has both outpatient and inpatient departments, with a bed capacity of 45. It also provides services to neighboring health centers, including Nyamirima, Nyakatokye, and Kyikuchu. The ART clinic operates four days a week (Tuesday, Wednesday, Thursday, and Friday) from 8 a.m. to 5 p.m., with over 150 patients being reviewed daily. The study involved adult PLWHIV who are on dolutegravir-based ART at Ruhoko Health Centre IV.

Selection criteria and sampling procedure 

PLWHIV aged 18 years and above who had been on dolutegravir-based ART for at least 12 months and had provided informed consent were enrolled. Individuals with missing clinical records, pregnant women, those on interrupted treatment, and those who visited due to an acute illness (including hepatitis) were excluded. Those who were on diabetes treatment, lipid-lowering medication, corticosteroids, or oral contraceptive pills were also excluded from the study. The study participants were selected using a systematic sampling of adult HIV-positive patients on dolutegravir-based ART attending the clinic.

Sample size determination

Kish Leslie's formula of 1965 [27] was used to determine the minimum required sample size for the prevalence of MetS and its association with AST/ALT ratio using the following assumptions: 22.9% proportion of HIV-infected patients on highly active ART with MetS at Bugando Medical Centre in Mwanza, Tanzania [28], 5% precision and a 95% confidence interval and a Z-statistic of 1.96.

n = Z2P(1-P)/d2,

where n is the required sample size, P is the hypothesized prevalence, Z is the Z-value for the confidence level, and d is precision.

n= 1.962 x 0.229(1-0.229)/0.052 = 227.1

With a 10% unresponsive rate, the minimum sample size was 298 participants.

For the predictive performance of the ALT/AST ratio, at an AUC (0.618) of ALT/AST > 1 in the prediction of MetS, the optimal cutoff determined by the Youden index is >0.852, with a sensitivity of 56.7% (95% CI 53.9-59.4%) and a specificity of 62% (95% CI 59.1-64.8%) [29], a single proportion formula by Cochrane was used to compute the required sample size using a sensitivity of 56.7%.

n = V2P(1-P)/d2,

where n is the required sample size, V = desired level of statistical significance, i.e., 1.96, P = sensitivity of 56.7% obtained from a similar study in southern Taiwan between March and December 2019 (29), and d = minimum clinically desired precision = 5%.

Therefore, n = 1.962 * 0.567(1-0.567)/0.052 = 377.

At a specificity of 62% (29), a sample size of 362 was determined.

Since the sample size calculation using sensitivity gave the highest number of participants, a minimum of 377 study participants were recruited for this study.

Study variables

Dependent Variable

MetS was the dependent variable. MetS was defined as fulfilling three or more of the following criteria: waist circumference (WC) >102 cm for men and >88 cm for women; triglycerides (TG) ≥150 mg/dL; HDL-c <40 mg/L for men and <50 mg/dL in women; BP >130/85 mmHg or hypertension treatment; and fasting plasma glucose (FBG) ≥110 mg/dL or being on diabetes treatment (International Diabetes Federation (IDF) Consensus worldwide definition, 2006).

Independent Variables

ALT/AST ratio was the major independent variable of this study. The other independent variables were categorized as sociodemographic, behavioral, clinical, and HIV- and/or ART-related factors. The sociodemographic factors included sex, age, religion, education level, employment status, and marital status. The behavioral factors included smoking status, alcohol consumption, and physical activity. Clinical factors included hypertension, family history of hypertension, diabetes mellitus, overweight, waist circumference, obesity, waist-to-height ratio, and waist-to-hip ratio. HIV- and ART-related factors included duration of HIV infection and DTG-based ART, viral load, and CD4 count.

Data collection

Data were collected after obtaining ethical approval from the Mbarara University of Science and Technology Research Ethics Committee on June 18, 2024 (approval no. MUST-2024-1575). The study lasted one and a half months from July 1 to August 15, 2024. The information on sociodemographic and behavioral factors was collected using a semi-structured questionnaire (see Appendix) during interactions with the study participants. Medical records were reviewed to gather data on the duration of HIV infection, recent viral load, duration of DTG-based ART, most recent CD4 count, history of diabetes mellitus diagnosis, medications, and adherence to ART.

The measurements of height and weight, required for calculating BMI, as well as waist/hip circumferences needed to calculate the waist-to-hip ratio and blood pressure, were performed by a clinician. A digital sphygmomanometer was used to measure blood pressure. Two blood pressure readings were recorded at five-minute intervals, and their mean value was taken as the BP. The weight and height of the participants were measured using a portable weight and height scale. A non-stretchable seca 201 ergonomic circumference measuring tape was used to measure the waist and hip circumferences. Two circumference readings were taken, and a third measurement was performed if the difference between the first two exceeded 3 cm. The waist and hip circumferences were recorded as the mean of the two or three measurements.

About 4 ml of venous blood sample was collected from each respondent who had fasted overnight, using fluoride-oxalate (gray top) and red top vacutainer vacuum bottles. Plasma and serum were separated within less than one hour after blood collection. The biochemical parameters (plasma glucose, serum total cholesterol, LDL-c, HDL-c, triglycerides concentration, AST, ALT, ALP, GGT, and serum electrolytes) were measured using the HumaStar 100 clinical chemistry analyzer (Human Diagnostic, Germany) at Ruhoko Health Centre IV Clinical Chemistry Laboratory, Ibanda Municipality. This instrument is designed to perform spectrophotometric measurements of analyte levels at specific set wavelengths using designated reagents. The analyzer had a throughput of 100 tests per hour. It automatically performed all sample and reagent pipetting, photometric measurements, incubation, and calculations.

The calibration of the HumaStar 100 chemistry analyzer was performed using AutoCal (EFILive, Auckland, New Zealand). Normal quality control samples, such as HumaTrol N, and pathological quality control samples, such as HumaTrol P, were run on the analyzer daily before the research samples were processed. The manufacturer’s instructions for the machine and the reagents were strictly followed. Ruhoko Health Centre IV Laboratory is a nationally audited laboratory at a level of star 2 by the National Health Laboratory Services/Central Public Health Laboratories (NHLS/CPHL), Ministry of Health, Uganda. In addition, 10% of the samples collected were tested at the Mbarara Regional Referral Hospital clinical chemistry laboratory to guarantee the accuracy of the results obtained from the onsite testing laboratory.

Statistical analysis

The data were analyzed using Stata Statistical Software (release 17, StataCorp LLC, College Station, TX). The distribution of variables was compared between the participants with and those without MetS at a univariate level to test for statistical differences between the two groups. Categorical variables were summarized using frequencies and proportions, and then a Chi-square test or Fisher’s exact test was used to compare their distribution between the two groups of the outcome variable. Mean ± SD was utilized to summarize continuous variables that were normally distributed, while median (IQR) was used to summarize those that were not normally distributed. A t-test was used to compare means between the dependent variable categories, while the Wilcoxon rank-sum test was used to compare the medians where the data were not normally distributed across the study participants.

The prevalence of MetS was expressed as a proportion. Logistic regression was used to assess the association between AST/ALT ratio and MetS. MetS was the binary dependent variable. All independent variables including the primary exposure variable (AST/ALT ratio), at the bivariate level, were compared with MetS. The associations were measured using odds ratios together with their 95% confidence intervals (CIs), and the statistically significant odds ratios were indicated by a p-value <0.05. The variables that were clinically and/or statistically significant at this level were assessed in the multivariable model to adjust for confounding effects. The Hosmer-Lemeshow test was used to test the suitability of the final multivariate model in predicting probable dementia. A p-value of 0.8259 was obtained indicating good goodness of fit for the final selected model. In the final multivariable model, associations were considered significant at a p-value <0.05. A receiver operating characteristic (ROC) curve was used to evaluate the performance of the ALT/AST ratio to predict MetS. The area under the curve (AUC) was used to assess the predictive power, with a value close to one showing better performance.

## Results

A total of 377 participants were recruited in this study. The median age of the study participants was 44 years (IQR: 30-59) with the majority being below 40 years (166, 44.0%). The majority of the study participants were female (212, 56.2%), and married/cohabiting 216 (57.3%), as indicated in Table 1. The proportion of other sociodemographic participant characteristics is indicated in Table 1. A total of 376 (99.7%) study participants were on TDF/3TC/DTG ART regimen, and 265 (70.3%) were on DTG-based ART for a period greater than two years (Table 1). Participants with MetS were observed to have a significantly lower AST/ALT ratio, i.e., 0.83 (IQR: 0.63-1.09) in comparison to those without MetS, which is 0.94 (IQR: 0.71-1.34), with p-value = 0.008. We also observed significant differences in the distribution of age, sex, education levels, smoking status, diagnosed hypertension, obstructive sleep apnea, family history of hypertension, DTG-based ART duration, mid-upper arm circumference (MUAC), body mass index (BMI), aspartate aminotransferase (AST), and chloride, with p-value <0.05 between participants with and those without MetS (Table 1).

**Table 1:** Characteristics of the study participants stratified by metabolic syndrome status Notes: Data has been presented as N (%) and medians (IQR). Proportions were compared using the chi-square test and Fisher's exact test. Medians were compared using the Wilcoxon rank-sum test. A p-value <0.05 was taken to be statistically significant. Abbreviations: ART, antiretroviral therapy; ALT, alanine aminotransferase; AST, aspartate aminotransferase; ALP, alkaline phosphatase; DTG, dolutegravir, GGT, gamma-glutamyl transferase; MUAC, ,id upper arm circumference, PSQI, Pittsburgh Sleep Quality Index; Na+, sodium ions; K+, potassium ions; Cl-, chloride ions

Variable	Total N = 377	Metabolic syndrome		Statistical test	p-value
		Absent N = 244	Present N = 133		
Age in years: Median(IQR)	44 (30-59)	39 (29-54)	48 (38-63)	Wilcoxon rank-sum	<0.001
Age(years)	-	-	-	Chi-square	<0.001
<40	166 (44.0%)	126 (51.6%)	40 (30.1%)	-	-
40-60	124 (32.9%)	76 (31.1%)	48 (36.1%)	-	-
>60	87 (23.1%)	42 (17.2%)	45 (33.8%)	-	-
Sex	-	-	-	Chi-square	<0.001
Male	165 (43.8%)	125 (51.2%)	40 (30.1%)	-	-
Female	212 (56.2%)	119 (48.8%)	93 (69.9%)	-	-
Marital status	-	-	-	Chi-square	<0.001
Single	105 (27.9%)	85 (34.8%)	20 (15.0%)	-	-
Married/cohabiting	216 (57.3%)	125 (51.2%)	91 (68.4%)	-	-
Separated/divorced	56 (14.9%)	34 (13.9%)	22 (16.5%)	-	-
Education level	-	-	-	Chi-square	<0.001
No education	83 (22.0%)	42 (17.2%)	41 (30.8%)	-	-
Primary	68 (18.0%)	40 (16.4%)	28 (21.1%)	-	-
Secondary	75 (19.9%)	47 (19.3%)	28 (21.1%)	-	-
Tertiary	151 (40.1%)	115 (47.1%)	36 (27.1%)	-	-
Residence	-	-	-	Chi-square	0.49
Urban	216 (57.3%)	143 (58.6%)	73 (54.9%)	-	-
Rural	161 (42.7%)	101 (41.4%)	60 (45.1%)	-	-
Religion	-	-	-	Chi-square	0.65
Catholic	112 (29.7%)	73 (29.9%)	39 (29.3%)	-	-
Protestant	138 (36.6%)	88 (36.1%)	50 (37.6%)	-	-
Moslem	32 (8.5%)	18 (7.4%)	14 (10.5%)	-	-
Others	95 (25.2%)	65 (26.6%)	30 (22.6%)	-	-
Employment status	-	-	-	Chi-square	0.26
Unemployed	60 (15.9%)	35 (14.3%)	25 (18.8%)	-	-
Employed	317 (84.1%)	209 (85.7%)	108 (81.2%)	-	-
Ventilated chicken	-	-	-	Chi-square	0.30
No	55 (14.6%)	39 (16.0%)	16 (12.0%)	-	-
Yes	322 (85.4%)	205 (84.0%)	117 (88.0%)	-	-
smoking	-	-	-	Chi-square	0.042
Nonsmoker	332 (88.1%)	221 (90.6%)	111 (83.5%)	-	-
Ever smoked	45 (11.9%)	23 (9.4%)	22 (16.5%)	-	-
Alcohol status	-	-	-	Chi-square	0.093
Never consumed	143 (37.9%)	85 (34.8%)	58 (43.6%)	-	-
Ever consumed	234 (62.1%)	159 (65.2%)	75 (56.4%)	-	-
Diagnosed hypertension	-	-	-	Chi-square	<0.001
No	315 (83.6%)	217 (88.9%)	98 (73.7%)	-	-
Yes	62 (16.4%)	27 (11.1%)	35 (26.3%)	-	-
Vegetable fruit intake	-	-	-	Fisher's Exact	0.50
<5 servings per day	367 (97.3%)	236 (96.7%)	131 (98.5%)	-	-
≥5 servings per day	10 (2.7%)	8 (3.3%)	2 (1.5%)	-	-
Global PSQL Score; Median(IQR)	4 (2-6)	4 (2-6)	4 (2-6)	Wilcoxon rank-sum	0.30
Obstructive Sleep Apnea	-	-	-	Chi-square	0.002
Low risk of OSA	302 (80.1%)	207 (84.8%)	95 (71.4%)	-	-
High risk of OSA	75 (19.9%)	37 (15.2%)	38 (28.6%)	-	-
Sleep duration	-	-	-	Chi-square	0.37
>7	72 (19.1%)	44 (18.0%)	28 (21.1%)	-	-
6-7	251 (66.6%)	162 (66.4%)	89 (66.9%)	-	-
5-6	38 (10.1%)	29 (11.9%)	9 (6.8%)	-	-
<5	16 ( 4.2%)	9 (3.7%)	7 (5.3%)	-	-
Family history of hypertension	-	-	-	Chi-square	0.005
No	314 (83.3%)	213 (87.3%)	101 (75.9%)	-	-
Yes	63 (16.7%)	31 (12.7%)	32 (24.1%)	-	-
Family history of diabetes mellitus	-	-	-	Chi-square	0.25
No	285 (75.6%)	189 (77.5%)	96 (72.2%)	-	-
Yes	92 (24.4%)	55 (22.5%)	37 (27.8%)	-	-
Family history of CVD	-	-	-	Fisher's Exact	0.072
No	368 (97.6%)	241 (98.8%)	127 (95.5%)	-	-
Yes	9 (2.4%)	3 (1.2%)	6 ( 4.5%)	-	-
Family history of dyslipidemia	-	-	-	Chi-square	0.85
No	361 (95.8%)	234 (95.9%)	127 (95.5%)	-	-
Yes	16 (4.2%)	10 (4.1%)	6 (4.5%)	-	-
Family history of kidney disease	-	-	-	Chi-square	0.97
No	315 (83.6%)	204 (83.6%)	111 (83.5%)	-	-
Yes	62 (16.4%)	40 (16.4%)	22 (16.5%)	-	-
ART duration	-	-	-	Chi-square	0.007
≤5 years	115 (30.5%)	86 (35.2%)	29 (21.8%)	-	-
>5 years	262 (69.5%)	158 (64.8%)	104 (78.2%)	-	-
DTG-ART duration	-	-	-	Chi-square	0.001
≤2 years	112 (29.7%)	86 (35.2%)	26 (19.5%)	-	-
>2 years	265 (70.3%)	158 (64.8%)	107 (80.5%)	-	-
MUAC (cm) median (IQR)	28 (27-29)	28 (27-29)	29 (27-31)	Wilcoxon rank-sum	<0.001
BMI (kg/m^2^) median (IQR)	22.72 (20.32-25.86)	22.28 (20.16-24.99)	23.74 (21.22-27.99)	Wilcoxon rank-sum	<0.001
Total cholesterol (mg/dL) median (IQR)	168 (144-208.8)	171 (147-210)	164 (138-206)	Wilcoxon rank-sum	0.14
AST (IU/L) median (IQR)	21 (15-30)	22 (16-31.6)	18 (14-28)	Wilcoxon rank-sum	0.016
ALT (IU/L) median (IQR)	23 (17-31)	23 (17-31)	23 (18-31)	Wilcoxon rank-sum	0.93
AST/ALT ratio median (IQR)	0.90 (0.66-1.28)	0.94 (0.71-1.34)	0.83 (0.63-1.09)	Wilcoxon rank-sum	0.008
ALP (IU/L) median (IQR)	101 (82-119)	101.45 (82-118)	101 (84-121)	Wilcoxon rank-sum	0.84
GGT (IU/L) median (IQR)	19 (13-29)	19 (13-29)	19 (14-27)	Wilcoxon rank-sum	0.97
Na^+ ^(mmol/L) median (IQR)	137.8 (135.40-141)	138.20 (135.5-141.2)	137.2 (135.40-140)	Wilcoxon rank-sum	0.12
K^+ ^(mmol/L) median (IQR)	4.03 (3.81-4.41)	4.07 (3.81-4.48)	4.01 (3.81-4.31)	Wilcoxon rank-sum	0.27
Cl^- ^(mmol/L) median (IQR)	99.40 (97.30-102.7)	100.2 (97.40-103.1)	98.70 (96.70-101.3)	Wilcoxon rank-sum	0.004

**Figure 1:**
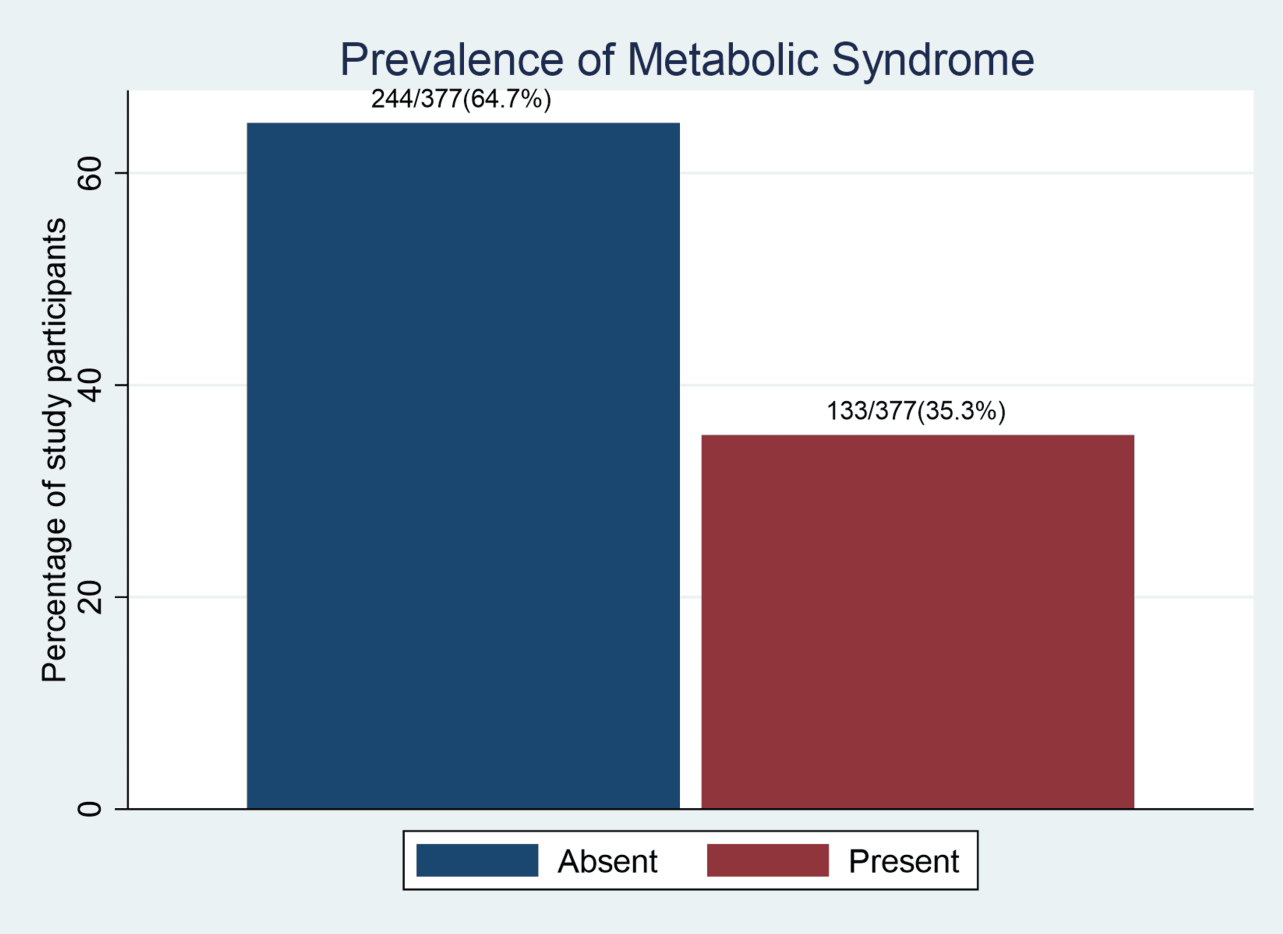
A bar graph showing the prevalence of metabolic syndrome among patients on dolutegravir-based antiretroviral therapy

Out of the 377 study participants, 133 (35.3%) met the definition of MetS. Therefore, our study's overall prevalence of MetS was 35.3 %( 95%CI: 30.6-40.3), as indicated in Figure 1 and Table 2. The prevalence of MetS was significantly higher among study participants with a low AST/ALT ratio of 93 /224(41.5%) in comparison to the prevalence observed among those with a high AST/ALT ratio of 40/153 (26.1%), with a p-value of 0.002. The prevalence of the individual MetS components is indicated in Table 2. The most prevalent MetS component was elevated triglycerides (265, 70.3%), followed by high blood pressure (232, 61.5%), low high-density lipoprotein levels (181, 48.0%), central obesity (99, 26.3%), and then fasting hyperglycemia (55, 14.6%).

**Table 2:** Metabolic syndrome and its components Data are presented as N (%).

Variable	Total N = 377
Metabolic syndrome	-
Absent	244 (64.7%)
Present	133 (35.3%)
High fasting blood glucose	-
Absent (<110 mg/dL)	322 (85.4%)
Present (≥110 mg/dL)	55 (14.6%)
Central obesity (high waist circumference)	-
Absent (<88 cm for female and <102 cm for male)	278 (73.7%)
Present (≥88 cm for female and ≥102 cm for male)	99 (26.3%)
Elevated triglycerides	-
Absent (<150 mg/dL)	112 (29.7%)
Present (≥150 mg/dL)	265 (70.3%)
Low high-density lipoprotein levels	-
Absent (≥50 mg/dL for female and ≥40 mg/dL for male)	196 (52.0%)
Present (<50 mg/dL for female and <40 mg/dL for male)	181 (48.0%)
High blood pressure (SBP ≥ 130 mmHg and/or DBP ≥ 85 mmHg)	-
Absent	145 (38.5%)
Present	232 (61.5%)

**Figure 2:**
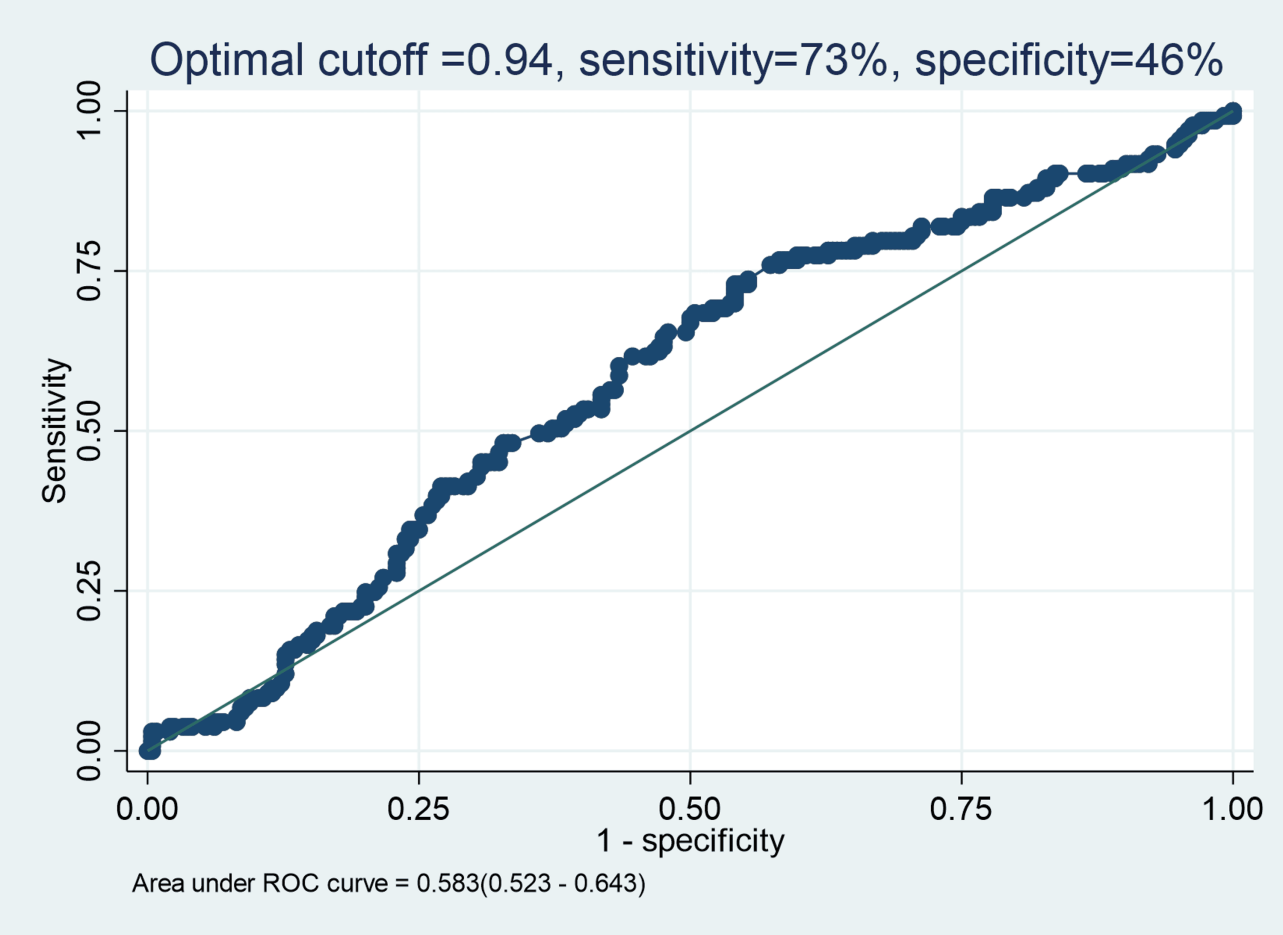
ROC curve showing the predictive performance of ALT/AST ratio for metabolic syndrome among patients on dolutegravir-based antiretroviral therapy Receiver operating characteristic (ROC) curve analysis was used to access the predictive performance. AST/ALT: aspartate aminotransferase-to-alanine aminotransferase

We observed a significant association between low AST/ALT ratio and MetS, i.e., aOR: 2.19 (95%CI: 1.28-3.73, p-value 0.004), as indicated in Table 3. Female gender (aOR: 3.68, 95%CI: 2.07-6.55, p-value <0.001) and smoking (aOR: 3.96, 95%CI: 1.77 - 8.86, p-value <0.001) were also found to have a significant association with MetS, as indicated in Table 3. We also identified a significant predictive performance of the ALT/AST ratio for MetS in our study population (AUC = 0.583, 95%CI: 0.523-0.643) in discriminating between those with and those without MetS, as indicated in Figure 2. At an optimal cut-off point of 0.94, the ALT/AST ratio was able to significantly distinguish participants with MetS from those without MetS at a sensitivity of 73% and specificity of 46%.

**Table 3:** Association between low AST/ALT ratio and metabolic syndrome Logistic regression analysis was used to assess association. Data are presented as OR (95% CI). AST/ALT: aspartate aminotransferase-to-alanine aminotransferase, cOR: crude odds ratio, aOR: adjusted odds ratio. A p-value <0.05 is statistically significant.

Variable	Bivariate analysis		Multivariate analysis	
	cOR (95%CI)	P-value	aOR (95%CI)	P-value
AST/ALT ratio	-	-	-	-
High (>)	1.00	-	1.00	-
Low (≤1)	2.01 (1.28 3.14)	0.002	2.19 (1.28 3.73)	0.004
Age (Years)	-	-	-	-
<40	1.00	-	1.00	-
40-60	1.99 (1.20 3.30)	0.008	1.74 (0.94 3.24)	0.079
>60	3.76 (1.95 5.85)	<0.001	1.96 (084 4.56)	0.118
Sex	-	-	-	-
Male	1.00	-	1.00	-
Female	2.44 (1.56 3.82)	<0.001	3.68 (2.07 6.55)	<0.001
BMI	-	-	-	-
<25 kg/m^2^	1.00	-	1.00	-
25-29 kg/m^2^	1.72 (1.05 2.84)	0.033	1.20 (0.65 2.21)	0.567
≥30 kg/m^2^	4.52 (2.01 10.16)	<0.001	1.75 (0.51 5.20)	0.375
Ventilated kitchen	-	-	-	-
No	1.00	-	1.00	-
Yes	1.39 (0.74 2.60)	0.300	1.39 (0.65 2.97)	0.394
Smoking	-	-	-	-
Nonsmoker	1.00	-	1.00	-
Ever smoked	1.90 (1.02 3.57)	0.044	3.96 (1.77 8.86)	<0.001
Alcohol status	-	-	-	-
Never consumed	1.00	-	-	-
Ever consumed	0.69 (0.45 1.07)	0.094	-	-
Diagnosed hypertension	-	-	-	-
No	1.00	-	-	-
Yes	2.87 (1.65 5.00)	<0.001	-	-
MUAC (cm)	1.17 (1.09 1.25)	<0.001	1.05 (0.95 1.17)	0.292
Vegetable fruit intake	-	-	-	-
<5 servings per day	1.00	-	1.00	-
≥5 servings per day	0.45 (0.09 2.15)	0.318	0.31 (0.05 1.20)	0.218
Poor sleep quality (Global PSQI score)	-	-	-	-
Good quality(≤5)	1.00	-	1.00	-
Poor quality(>5)	1.19 (0.74 1.91)	0.465	0.64 (0.32 1.29)	0.212
Obstructive sleep apnea	-	-	-	-
Low risk of OSA	1.00	-	1.00	-
High risk of OSA	2.24 (1.34 3.74)	0.002	2.15 (0.93 4.94)	0.073
Sleep duration (Hours)	-	-	-	-
>7	1.00	-	1.00	-
6-7	0.86 (0.50 1.48)	0.594	01.08 (0.56 2.07)	0.811
5-6	0.47 (0.20 1.18)	0.112	0.69 (0.24 2.00)	0.496
<5	1.22 (0.41 3.66)	0.720	1.43 (0.37 5.59)	0.603
Family history of hypertension	-	-	-	-
No	1.00	-	1.00	-
Yes	2.18 (1.26 3.76)	0.005	1.91 (0.85 4.33)	0.119
Family history of diabetes mellitus	-	-	-	-
No	1.00	-	1.00	-
Yes	1.32 (0.82 2.15)	0.255	0.73 (0.38 1.41)	0.347
Family history of cardiovascular diseases	-	-	-	-
No	1.00	-	-	-
Yes	3.80 (0.93 15.43)	0.062	-	-
Family history of dyslipidemia	-	-	-	-
No	1.00	-	-	-
Yes	1.11 (0.39 3.11)	0.849	-	-
Family history of kidney disease	-	-	-	-
No	1.00	-	-	-
Yes	1.01 (0.57 1.79)	0.970	-	-
ART duration	-	-	-	-
≤5 years	1.00	-	-	-
>5 years	1.95 (1.20 3.18)	0.007	-	-
DTG-ART duration	-	-	-	-
≤2 years	1.00	-	1.00	-
>2 years	2.24 (1.36 3.70)	0.002	1.43 (0.75 2.72)	0.277
Sodium categories (mmol/L)	-	-	-	-
135-145	1.00	-	1.00	-
<135	0.99 (0.61 1.64)	0.993	0.78 (0.43 1.41)	0.404
>145	0.41 (0.16 1.04)	0.062	0.47 (0.15 1.43)	0.183
Potassium categories (mmol/L)	-	-	-	-
3.5-4.5	1.00	-	1.00	-
<3.5	0.85 (0.41 1.78)	0.665	0.72 (0.29 1.79)	0.480
>4.5	0.56 (0.32 1.00)	0.050	0.61 (0.32 1.19)	0.146
Chloride (mmol/L)	-	-	-	-
96-106	1.00	-	1.00	-
<96	1.29 (0.74 2.26)	0.369	1.56 (0.81 3.03)	0.187
>106	0.24 (0.07 0.83)	0.025	0.19 (0.05 0.74)	0.017
Alkaline phosphatase (ALP)	-	-	-	-
≤130U/L	1.00	-	1.00	-
>130 U/L	1.27 (0.75 2.14)	0.376	1.38 (0.73 2.60)	0.316
Gamma-glutamyl transferase (GGT)	-	-	-	-
≤40 U/L	1.00	-	1.00	-
>40 U/L	1.45 (0.68 3.08)	0.338	1.36 (0.54 3.42)	0.517
Total cholesterol	-	-	-	-
<200 mg/dL	1.00	-	1.00	-
≥200 mg/dL	0.89 (0.55 1.41)	0.610	0.61 (0.34 1.09)	0.096

## Discussion

This study identifies a significant association between MetS and the AST/ALT ratio among HIV-infected patients on dolutegravir-based ART at Ruhoko Health Centre IV in Uganda. The high prevalence of MetS observed in this study (133, 35.3%) aligns with previously reported data, indicating a substantial burden of MetS among HIV-infected individuals on ART, especially those on dolutegravir-based regimens. A high prevalence of MetS components such as hyperglycemia (33.3% [9] and 19.8% [30]), hypertension (27.2%) [9], dyslipidemia(78.0%) [31], and excessive weight gain (7.3%) [32] have already been reported among HIV-infected patients on dolutegravir-based ART in Uganda. Our study’s high prevalence is in line with the documented high prevalence (58%) of MetS among PLWHIV on ART (but not on DTG) in Southwestern Uganda [33].

Compared to other regional studies, our findings reveal a higher prevalence of MetS. For example, the 13.9% prevalence of MetS observed in HIV-infected patients before the initiation of dolutegravir-based ART in Kampala [34] highlights the potential impact of dolutegravir on the development of MetS. Similarly, studies from Tanzania [28,35] and Ethiopia [36] reported lower prevalence rates of MetS compared to our findings, suggesting a specific influence of Dolutegravir on metabolic health.

The MetS prevalence documented in our study was also significantly higher than the prevalence (29.5%) of MetS documented in a study conducted in the Sidama Region, South Ethiopia [8]. Our study population was HIV-infected participants on dolutegravir-based ART, a drug regimen that has been associated with a high prevalence of components of MetS such as weight gain, hyperglycemia, hypertension, and dyslipidemia. This probably explains the higher prevalence documented in our study than in the Ethiopia study where the study population was not necessarily on dolutegravir-based ART.

The significant association between a low AST/ALT ratio and MetS in our study concurs with findings from Ethiopia [36], Mexico [37], Korea [22], and India [38], where lower AST/ALT ratios were noted among participants with MetS. These findings are significant because they suggest that the AST/ALT ratio could be a potential early indicator of MetS, which is critical for the early intervention and management of cardiometabolic risks in HIV patients on ART.

Moreover, the observed association between female gender and MetS in our study aligns with findings from Kampala, Uganda [34], Ethiopia [36], and South Africa [39]. This gender disparity may be attributed to hormonal differences, body fat distribution, and other socio-cultural factors influencing lifestyle and healthcare access.

The study also highlights the role of smoking as a risk factor for MetS, echoing previous research that links smoking to insulin resistance and central fat accumulation [40]. This association agrees with a study conducted among adult men in Korea [41]. This finding emphasizes the importance of integrating smoking cessation programs into the care of HIV patients on ART to reduce the risk of MetS and related complications.

The predictive performance of the AST/ALT ratio for MetS, as indicated by the AUC analysis, suggests that the AST/ALT ratio is a useful biomarker for identifying patients at risk of developing MetS. This agrees with similar findings in Taiwan, where the ALT/AST ratio showed good predictive ability for MetS [29].

However, our cross-sectional study design limits our ability to infer causality between the AST/ALT ratio and MetS. In addition, the reliance on self-reported fasting status may have introduced bias to the determination of fasting glucose and triglycerides. Future studies should consider a longitudinal design to better assess the temporal relationship between liver enzymes and MetS development.

## Conclusions

This study provides compelling evidence indicating a high prevalence of MetS among HIV-infected individuals on dolutegravir-based ART. Low AST/ALT ratio, female gender, and smoking are significantly associated with MetS. ALT/AST ratio demonstrated considerable discriminative power in distinguishing between individuals with MetS and those without. These findings suggest that a low AST/ALT ratio may serve as a valuable biomarker for identifying MetS in this population.

## Figures and Tables

**Figure 3:**
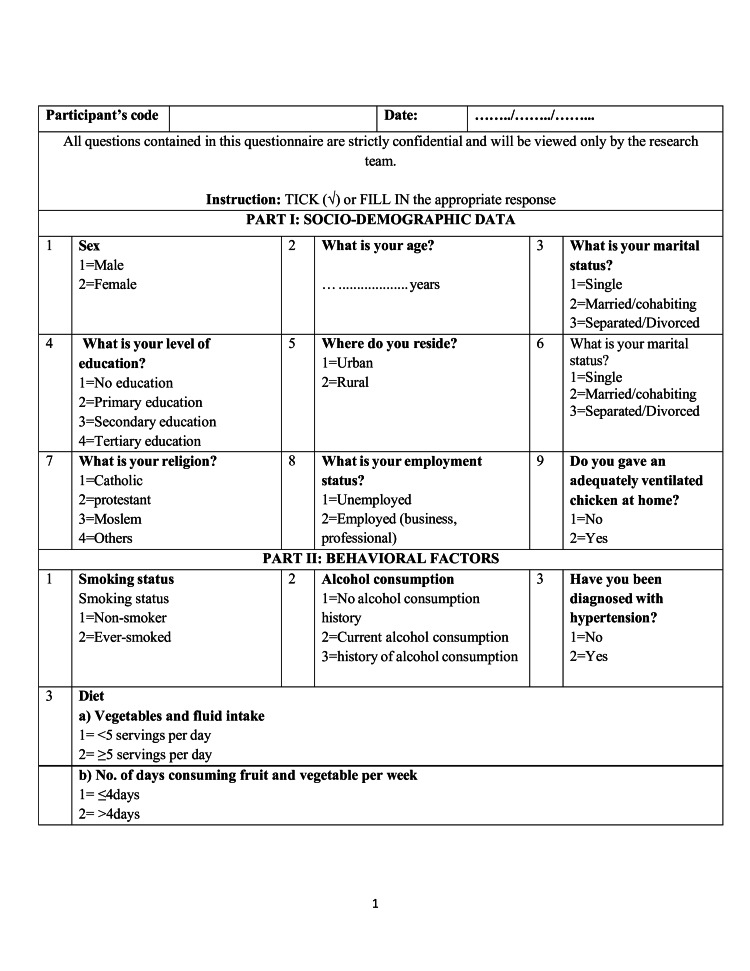
Sociodemographic data and behavioral factors

**Figure 4:**
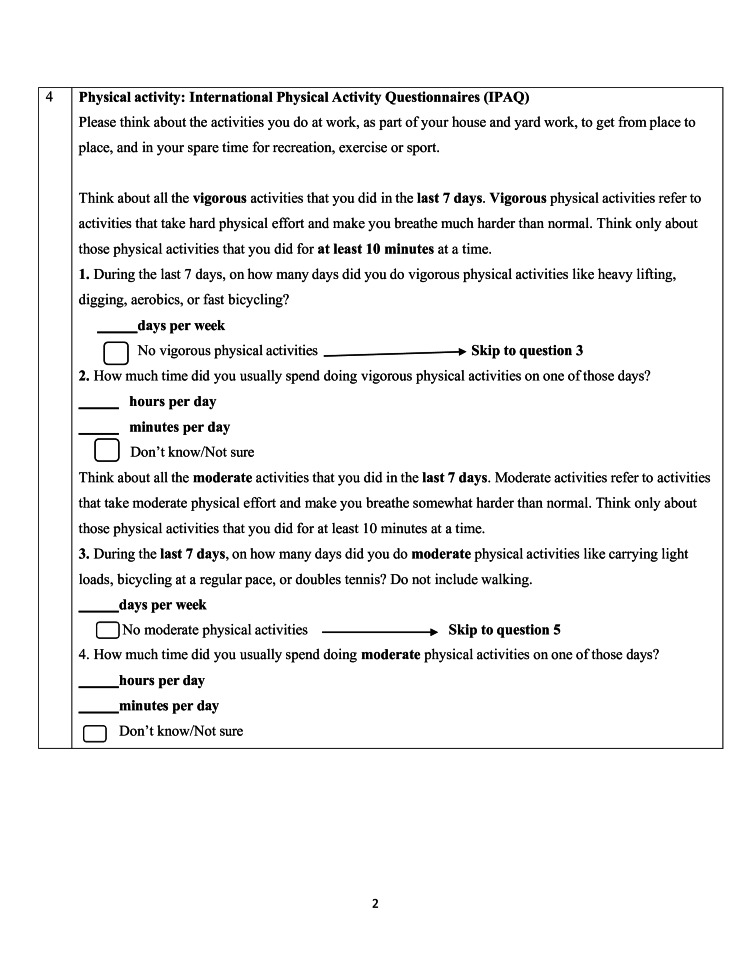
International physical activity questionnaires (IPAQ) - part 1

**Figure 5:**
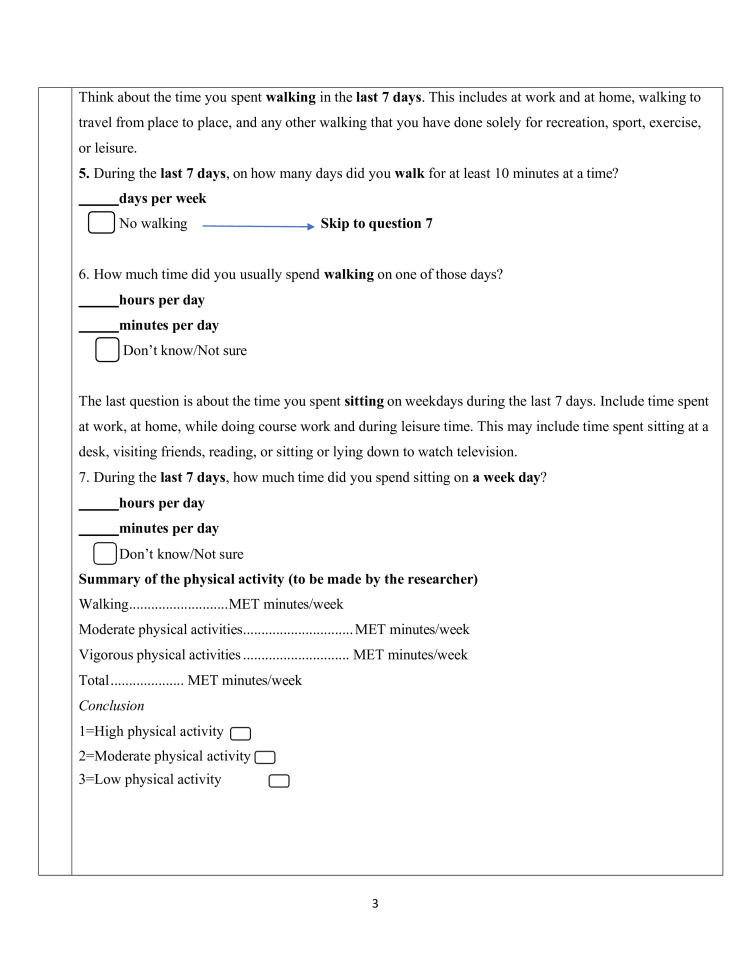
International physical activity questionnaires (IPAQ) - part 2

**Figure 6:**
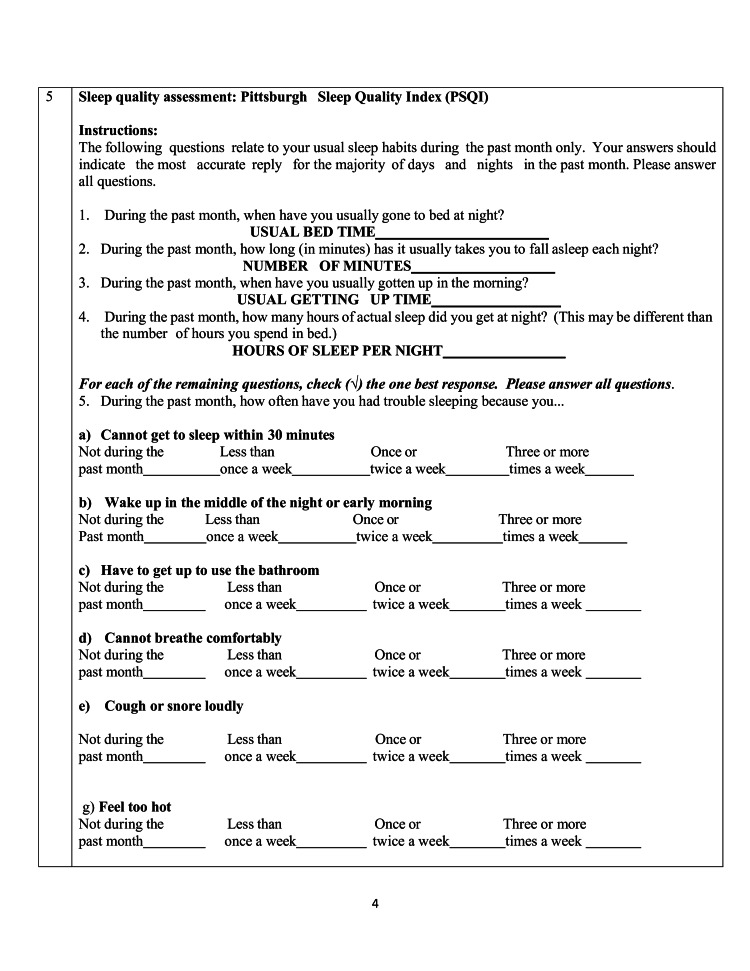
Sleep quality assessment: Pittsburgh Sleep Quality Index (PSQI) - part 1

**Figure 7:**
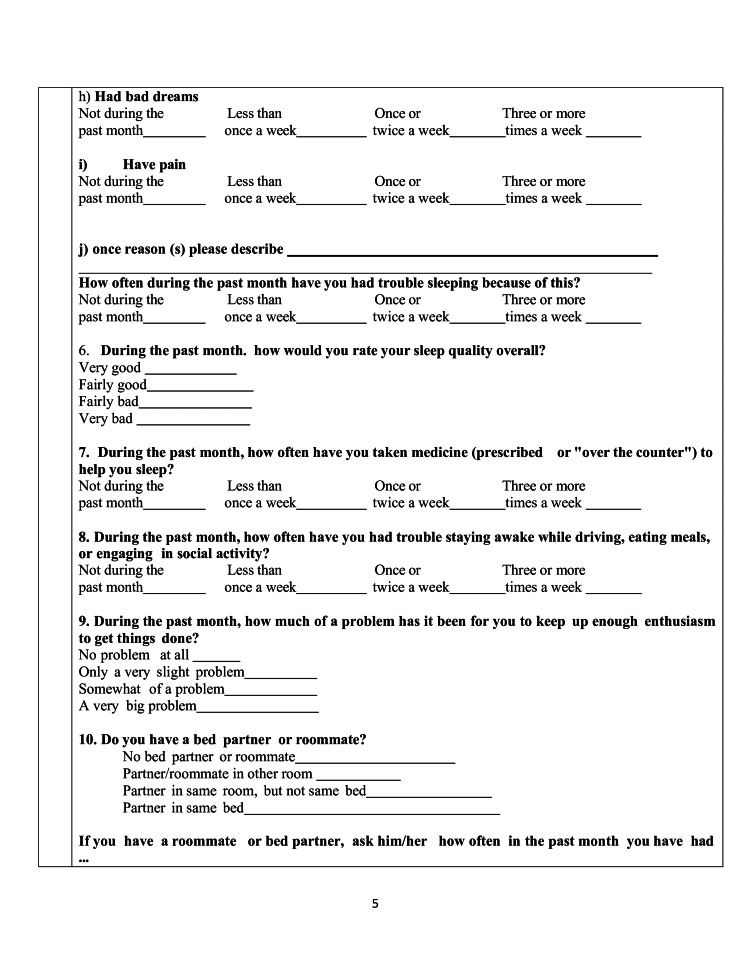
Sleep quality assessment: Pittsburgh Sleep Quality Index (PSQI) - part 2

**Figure 8:**
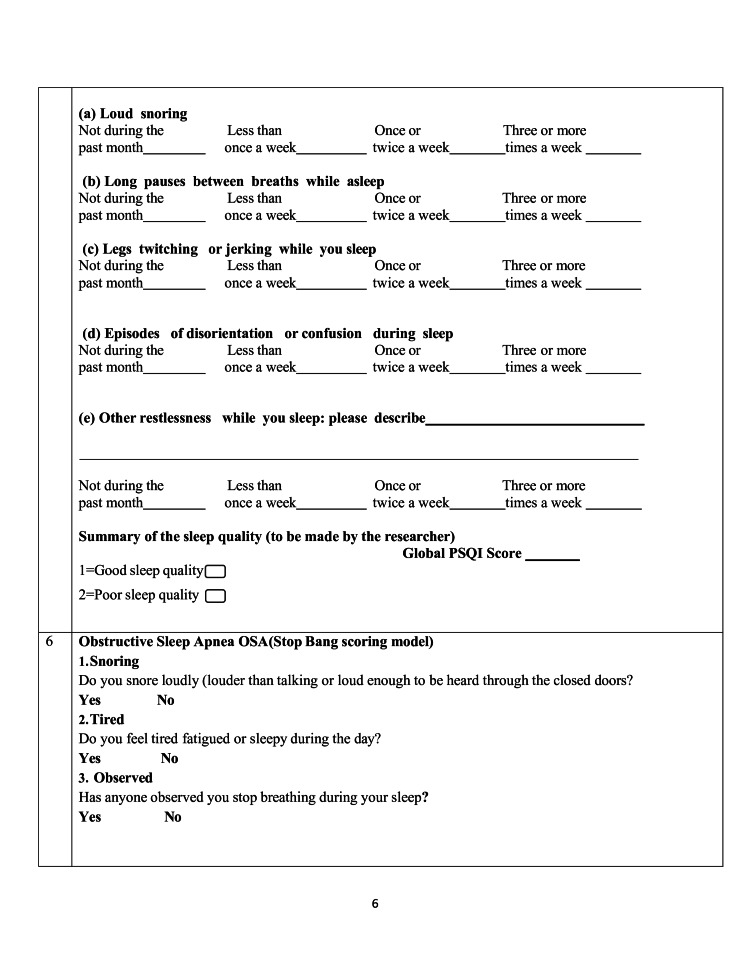
Sleep quality assessment: Pittsburgh Sleep Quality Index (PSQI) part 3 and obstructive sleep apnea part 1

**Figure 9:**
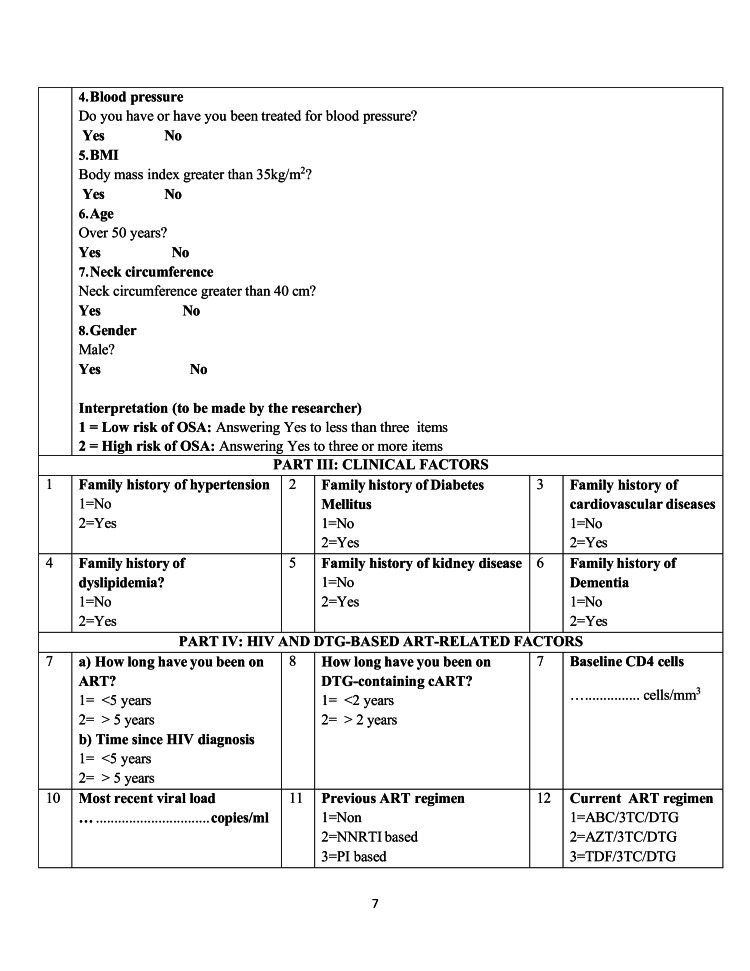
Obstructive sleep apnea (part 2) and clinical and HIV-related factors

**Figure 10:**
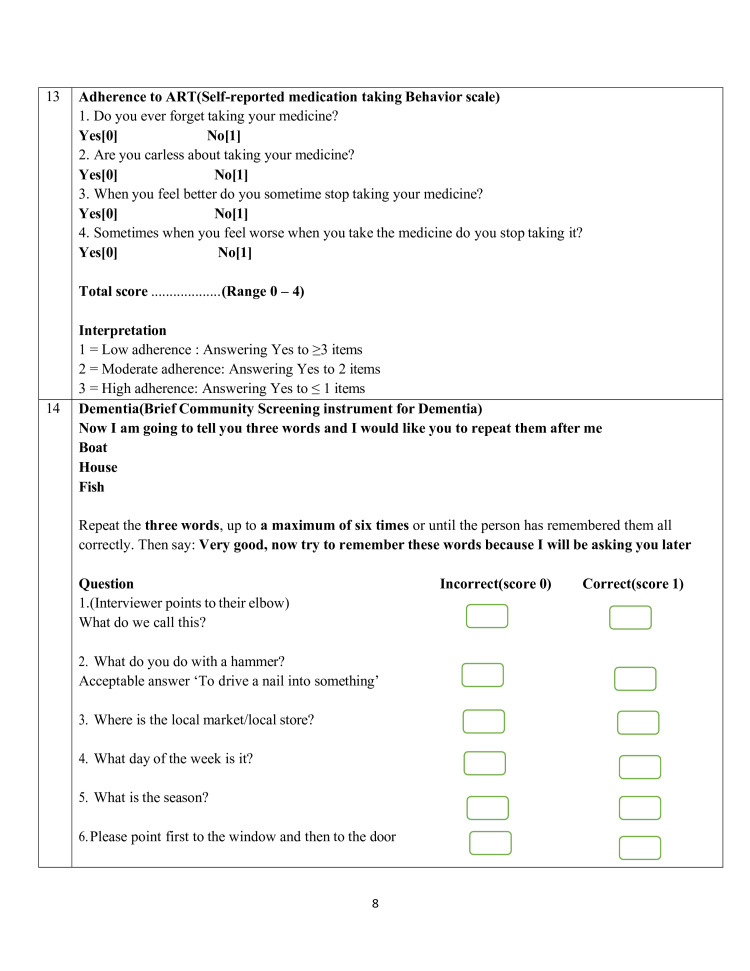
Adherence to antiretroviral therapy (ART) and dementia (brief community screening instrument for dementia) - part 1

**Figure 11:**
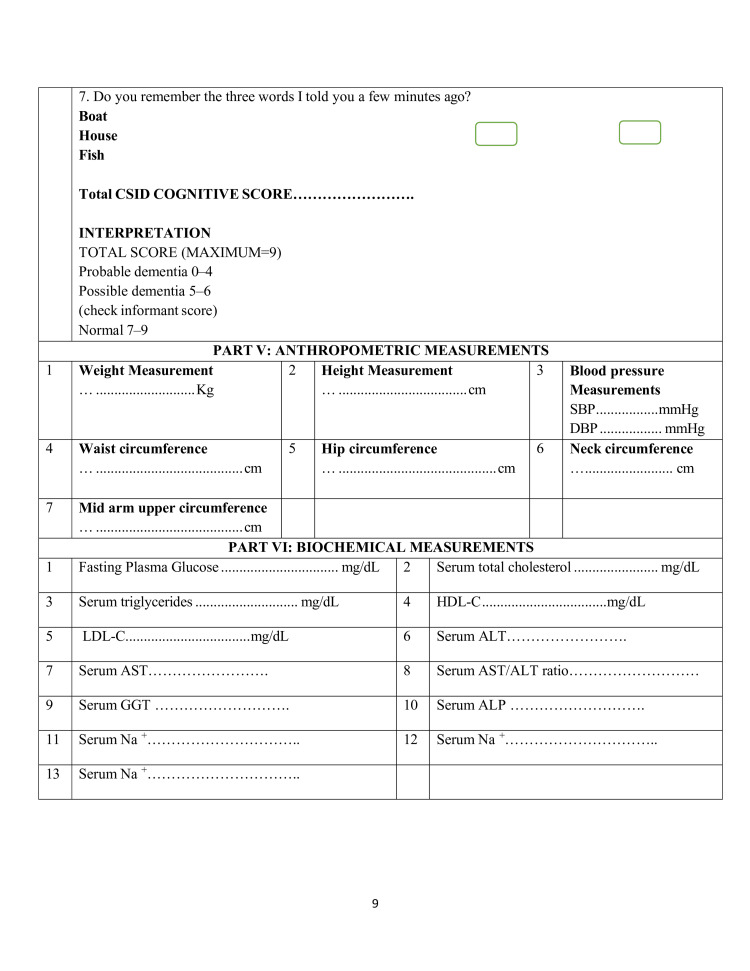
Dementia (part 2): anthropometric and biochemical measurements

## Appendices

Study questionnaire
